# Ultrasonography Is Not Inferior to Fluoroscopy to Guide Extracorporeal Shock Waves during Treatment of Renal and Upper Ureteric Calculi: A Randomized Prospective Study

**DOI:** 10.1155/2017/7802672

**Published:** 2017-05-15

**Authors:** Jeroen Van Besien, Pieter Uvin, Isabeau Hermie, Thomas Tailly, Luc Merckx

**Affiliations:** ^1^Department of Urology, AZ Sint-Lucas, Ghent, Belgium; ^2^Department of Radiology, AZ Sint-Jan, Bruges, Belgium; ^3^Department of Urology, University Hospitals, Ghent, Belgium

## Abstract

**Objective:**

To investigate whether the visualization modality (ultrasound or fluoroscopy) used during shockwave lithotripsy (SWL) affects the clinical outcome in those instances where both imaging modalities are optional.

**Methods:**

Between November 2014 and July 2016, 114 patients with radiopaque upper urinary tract calculi were randomly assigned to an ultrasound- or fluoroscopy-guided SWL group in a prospective, open-label, single-center study. A standardized SWL protocol was used. The stone-free rate and the positive outcome rate (stone-free or asymptomatic residual fragments ≤ 4 mm) were compared.

**Results:**

The stone-free rate was 52% in the ultrasound-guided group compared to 42% in the fluoroscopy-guided group (*p* = 0.06) and the positive outcome rate was 79% in the ultrasound-guided group compared to 70% in the fluoroscopy-guided group (*p* = 0.28). These results were not significantly different but proved to be noninferior based on a Wilson confidence interval of independent proportions (noninferiority limit 10%). The mean number of SWL sessions was not significantly different (*p* = 0.4).

**Conclusion:**

Our study demonstrated that the clinical results of ultrasound-guided SWL were not inferior to the results of fluoroscopy-guided SWL, while no ionizing radiation is needed.

## 1. Introduction

Extracorporeal shock wave lithotripsy (SWL) is a well known technique that has been used since the early eighties for the treatment of urinary stones [1]. Currently, there is a trend towards the preferred use of minimally invasive endoscopic procedures such as (flexible) ureteroscopy or (mini-) percutaneous nephrolithotomy for the treatment of renal or pyelum stones. Despite this evolution, SWL remains one of the preferred treatment options for renal stones < 20 mm [2]. SWL has a low complication rate and does not require general anesthesia [3].

The success rates for SWL vary strongly, as stone-free rates for renal and ureteric stones of 32–90% and 43–98%, respectively, have been reported [4]. The success rate depends on several patient and stone related factors, as well as treatment protocol and operator experience [4–9].

It is crucial for the success of SWL to correctly visualize the stone in order to focus the shock waves as precisely as possible, which is done by ultrasonography (US) (B–scan ultrasound) or fluoroscopy (FS) (X-rays). Radiopaque stones located in the kidney calices, the renal pelvis, or the ureteropelvic junction (UPJ) can often be visualized by both US and FS. In this paper these stones will be named upper urinary tract stones (UUTS). To the best of our knowledge, a head-on comparison of FS-guided versus US-guided SWL has not been reported in the published literature.

This study aimed to investigate whether the visualization modality affects the clinical outcome of SWL in those stones where we can choose between both imaging modalities (US and FS).

## 2. Materials and Methods

**Figure 1:**
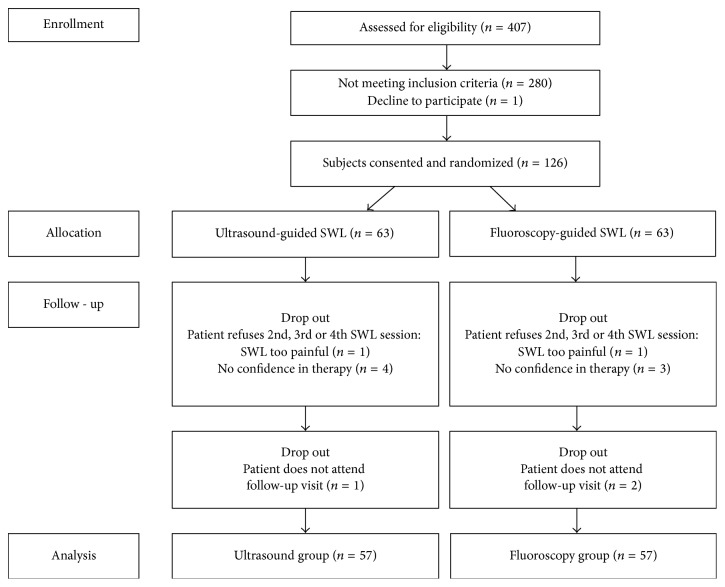
Outline of enrollment.

This study was approved by the institutional review board of Sint-Lucas Hospital Ghent and all patients signed an informed consent form. Outline of enrollment is depicted in Figure 1. Indications and exclusion criteria for SWL were according to the 2015 EAU guidelines on urolithiasis [2]. The study was performed between November 2014 and July 2016. Patients with radiopaque UUTS were eligible to be enrolled in this prospective single-center study. Prior to the treatment, all patients were randomized to an US-guided or a FS-guided SWL group based on a randomized list generated by statistical software. If a stone was not visible on abdominal radiograph and ultrasound, the patient was excluded from the study. The radiologist was blinded for the randomization process. Practitioner and patient were not. These SWL sessions have been carried out by only two practitioners. Both practitioners were used to performing renal ultrasonography in daily practice and had performed multiple US-guided and FS-guided SWL procedures prior to the start of the study. Their experience level with both techniques was equal.

Prior to the treatment, the size of the stone was measured by the radiologist by plain abdominal radiography using the maximal stone diameter [10]. The locations of the stones in the urinary tract were described as upper pole stones, midpolar stones, lower pole stones, or renal pelvis/UPJ stones. Sixty out of 114 patients that completed the study received a low dose noncontrast CT scan of the abdomen (5 mSv). In these patients, stone density (measured by mean and maximal Hounsfield units) and skin-to-stone distance (45° and 90°) were noted. SWL was performed on ambulatory basis using a diclofenac 100 mg sustained release suppository for analgesia [11]. SWL was performed with a 3rd generation Dornier Compact Sigma (Dornier MedTech, Munich, Germany). Fluoroscopic visualization was performed with a mobile digital C-Arm (Series 7700 OEC Medical Systems, General Electric, Boston, United States). Ultrasonographic visualization was done with a mobile ultrasonography machine (Flexfocus 400 BK Medical, Analogic, Boston, United States). The ultrasonographic visualization allowed real time monitoring of the SWL procedure by using hit/miss monitoring through spectral Doppler ultrasound [12].

The SWL protocol was standardized. The stone was positioned in the expiratory phase [13]. SWL was applied with patients in the supine position with a frequency of 1 Hz. A specific and standardized ramping protocol was applied. Energy was started at the lowest energy level possible with the SWL device. After 60-120-180-360 and 600 shocks the energy level was raised by one level. If the patient could not endure the discomfort created by the shock wave, the energy was lowered by one level for 120 shocks before attempting to raise the energy level again. At the end of the procedure, a maximal total shock wave energy of 62 Joule could be obtained in a patient that endured the ramping protocol completely. The duration of the positioning of the patient was measured using a timer. This timer started from the moment the patient was positioned on the treatment table until the first shock was given.

For the fluoroscopy group the window was collimated to a 7 × 7 cm window after focusing of the stone. Intermittent fluoroscopy was used. Positioning was adjusted at the beginning of the SWL session and after every 300 shocks or when patients moved. The number of position changes was noted. If disintegration of the stone was visible, the energy level was not raised in this group. Dose area product (DAP) and fluoroscopy time was noted after each session.

For the ultrasonography group, positioning was adjusted at the beginning of the SWL session and further based on real time monitoring of the stone localization. The number of position changes was noted.

After the SWL session, patients were discharged and were instructed to void in a stone sieve and to take once daily tamsulosin 0.4 mg until the next visit [14]. Two or three weeks after every SWL session, follow-up imaging (ultrasound and plain abdominal radiography) was performed by a radiologist. Residual stone size was estimated on plain abdominal radiography.

**Figure 2:**
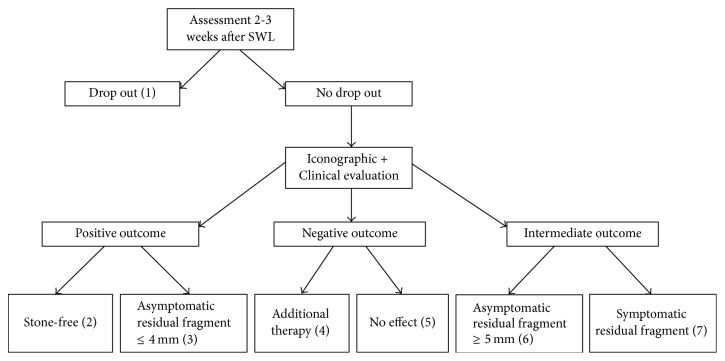
Seven possible outcomes after SWL sessions. (1) Drop out: patients refusing to further participate in the study or not attending follow-up visit after maximally 3 weeks (2) Positive endpoint: Stone-free patients. Follow-up visit after 1 year. (3) Positive endpoint: Asymptomatic residual fragments ≤ 4 mm. Follow-up visit after 6 months. (4) Negative endpoint: Additional therapy needed. Ureterorenoscopy, percutaneous nephrolithotomy, placement of an ureteric stent, or SWL on an ureteric stone no longer visible by ultrasound because of the location in the ureter. (5) Negative endpoint: No effect. No iconographic reduction in stone size and no gravel in the urine after 2 SWL sessions. (6) Intermediate outcome: Asymptomatic residual fragments ≥ 5 mm. Patients were proposed a second, third, or maximally fourth SWL session. (7) Intermediate outcome: Symptomatic residual fragments. Patients were proposed a second, third, or maximally fourth SWL session.

After each SWL session there were 7 possible outcomes, which are depicted in Figure 2. The primary outcome of this study was the number of patients with a stone-free status or the presence of only asymptomatic residual fragments of 4 mm or less after a maximum of four SWL sessions. This was defined as a* positive outcome* [15]. The absence of any result after two SWL sessions or the need for supplementary therapy was defined as a* negative outcome*. Declining further participation in the study or not attending follow-up consultation after SWL was defined as* drop out*. Secondary outcomes were the number of SWL sessions needed, positioning time, number of repositions, and dose area product.

All stone fragments were sent for examination by Fourier transform infrared spectroscopy. All patients were offered a further metabolic evaluation with a 24-hour urine collection.

Prior to the start of the study, a statistical power analysis for noninferiority studies was performed. Sample size estimation was based on our own retrospective data. To maintain 80% power with a significance level of 5% and a noninferiority level of 10% the projected sample size needed with this effect size was 57 patients per group. Adjusting for 10% loss to follow-up we recruited 126 patients. Noninferiority for the primary outcome was tested based on a 95% Wilson confidence interval for 2 independent proportions with the noninferiority limit set at 10%. Mann–Whitney *U* and unpaired *T*-test were performed for secondary outcomes where appropriate, considering a *p* value of <0.05 to indicate statistical significant difference. Statistical analysis was performed with IBM SPSS Statistics version 23.

## 3. Results

### 3.1. Factors Influencing SWL Outcome

One hundred and fourteen patients participated in this study. Both the US group and the FS group contained 57 patients. Most factors that could potentially influence the SWL outcome were not significantly different in both groups (Table 1). The mean* stone size* was 9 mm in the US group versus 8.5 mm in the FS group (*p* = 0.29). Only 55 patients (48%) were able to catch a fragment in a stone sieve. Thirty stone analyses were performed in the US group and 25 in the FS group. The US group harboured 6 “very hard” stones (Calcium Oxalate Monohydrate, brushite, or cystine stones [16]) compared to 4 in the FS group (*p* = 0.50). The position of the stones in the urinary tract differed (although not significantly) between both groups but the number of lower pole stones (which are known for their limited positive outcome after SWL [17]) was 22 in both groups. Mean and maximal stone densities were not significantly different in both groups (*p* = 0.10). The mean and maximal stone densities were 665 HU and 965 HU in the US group versus 740 HU and 1213 HU in the FS group. Due to the standardized study protocol, shock frequency, number of shocks, and ramping protocol were identical in both groups. Despite randomization, mean BMI and skin-to-stone distances were significantly higher in the FS group (*p* = 0.001 for BMI and *p* < 0.001 for skin-to-stone distances). The mean total energy used during an SWL session was significantly higher in the FS group compared to the US group (53 Joule compared to 49 Joule,* p* = 0.002).

### 3.2. Comparison of the Clinical Outcome

The* stone-free rate* was 52% (34/57) in the US-guided group (95% confidence interval 39.9%–65%) compared to 42% (24/57) in the FS-guided group (95% confidence interval 30.1%–55%) (Table 1). Superiority was tested with a Chi–Square test for independent data. The stone-free rate was not significantly different in both groups (*p* = 0.06). The success rate was estimated to be 10% (confidence interval 7.8% to 28.2%) higher for the US-guided group compared to the FS-guided group. The confidence interval contained value zero thus noninferiority of ultrasound was proven.

The positive outcome rate (stone-free or asymptomatic residual fragments) was 79% (45/57) in the US-guided group (confidence interval 66.7%–87.5%) compared to 70% (40/57) in the FS-guided group (confidence interval 57.3%–80.5%) (Table 1). Superiority was tested with a Chi–Square test for independent data. The positive outcome rate was not significantly different in both groups (*p* = 0.28). The success rate was estimated to be 9% (confidence interval 7.4% to 24.6%) higher for the US-guided group compared to the FS-guided group. The confidence interval contained value zero thus noninferiority of ultrasound was proven.

### 3.3. Total Number of SWL Sessions

The mean* number of SWL sessions* was 1.6 (91 SWL sessions/57 patients) in the US-guided group compared to 1.7 (97 SWL sessions/57 patients) in the FS-guided group and was not significantly different (*p *= 0.4).

### 3.4. Positioning Time and Position Adjustments

The mean time to position patients on the SWL table was significantly different between the two groups (*p* < 0.001) with 9 minutes in the US group compared to 5 minutes in the FS group (Table 1).

Despite a fixed protocol to reposition patients the* mean number of position changes* was 8 in the US group compared to 6 in the FS group (*p* = 0.008) (Table 1).

### 3.5. Radiation Exposure

We were able to visualize all the stones of the patients that were randomized with the allocated visualization method. Mean* dose area product* was 3005 mGy/cm^2^/SWL session in the FS group. Mean fluoroscopy time was 178 seconds. No radiation was used during the SWL procedures in the US group (Table 1).

## 4. Discussion

Radiopaque stones in the upper urinary tract can often be visualized both by ultrasonography and fluoroscopy during SWL treatment. Not all urologists have an ultrasound system that can be coupled to their SWL machine or they are not familiar or experienced with this ultrasound-guided SWL technique. The use of fluoroscopy is easier and may save some time. In our study, the mean time to position a patient in the US group was almost double the time to position a patient in the FS group. The mean difference however was only 4 minutes which is very limited, especially when the complete duration of an SWL procedure (up to one hour) is taken into account. This prospective study was performed with the intention to compare the results of SWL when both US and FS are possible. The study was intentionally limited to proximal ureter and renal calculi, since calculi in lower positions may be difficult to visualize with ultrasonography.

A major advantage of ultrasound is that it allows real time monitoring of the SWL procedure by using hit/miss monitoring through spectral Doppler ultrasound (SDU) [12]. Although theoretically this SDU technique could lead to a more accurate aiming of the shock wave because of the auditive and visual feedback, it is unknown whether this technical advantage also has a clinical advantage. To our knowledge, this study is the first to prospectively compare the clinical results of SWL guided with US (with real time monitoring) and fluoroscopy (with intermittent fluoroscopy).

The clinical results after SWL in our study showed that the chance of having a stone-free status or a positive outcome after US-guided ESWL treatment was slightly higher than after FS-guided SWL treatment (Table 1). A possible explanation for this difference might be the real time monitoring which led to a significantly higher number of repositioning in the US group. The difference, however, was not statistically significant. Using a noninferiority design, our study was able to prove that the noninferiority limit of 10% was easily met. This means that the clinical results of US-guided SWL were not inferior to FS-guided SWL. The mean number of SWL sessions needed to reach an endpoint, a secondary outcome, was not significantly different in both groups. Based on these results, we can only conclude that US is not inferior to FS.

Another important advantage of ultrasound is that it does not use* ionizing radiation*. In our study the mean fluoroscopy time was 178 seconds and the mean DAP was 3005 mGy/cm2/SWL session. Although this is not a worrisome dose compared to other interventional procedures, we need to bear in mind the cumulative effect of ionizing radiation. Patients may undergo more than one SWL session for the same stone. They might experience multiple stone episodes and multiple diagnostic imaging studies before and between SWL procedures. Imaging studies may also be performed for other purposes. Also, the operator's exposure to ionizing radiation should be taken into account [18]. Interestingly, however, Ordon and colleagues demonstrated that a small but significant increase in fluoroscopy time correlated with an increase in SWL success rates [19].

In our center, about 1/3rd of all stones for which SWL treatment is indicated can be visualized both by ultrasonography and fluoroscopy and in these cases we currently prefer to avoid radiation by using ultrasonography.

The major limitation of our study is the relatively small number of patients. A second limitation is the significant difference in BMI, skin-to-stone distances, and energy used during an SWL procedure between both groups despite randomization. It is known that a higher BMI and skin-to-stone distance may have a negative effect on SWL success [7, 8]. On the other hand, an insufficient energy level may have a negative effect on SWL success in the US group if the threshold energy level to achieve an appropriate stone disintegration is not reached [4]. It remains unknown to what extent these differences bias our study results considering the numerous factors that influence the clinical outcome of SWL.

The randomized prospective nature of the study and the fact that all SWL sessions were carried out with a standardized protocol on a single SWL machine confirm the strength of our study.

## 5. Conclusion

Our study demonstrates that US-guided SWL is not inferior to fluoroscopy-guided SWL, with the additional advantage of avoiding ionizing radiation. Although more research is needed to substantiate our findings, we would suggest to perform ultrasound-guided SWL whenever possible.

## Figures and Tables

**Table 1:** Summary of study results.

	US group (*n* = 57)	FS group (*n* = 57)	Statistical analysis
*Factors influencing SWL outcome*			

Stone-related factors			
*Stone size*			
Mean stone size (mm)^(1)^	9 (*σ* = 3)	8.5 (*σ* = 3)	*p* = 0.29
Number of stones 0–4 mm	5	5	*p* = 1
Number of stones 5–9 mm	30	30	*p* = 1
Number of stones 10–20 mm	22	22	*p* = 1
*Stone composition* ^*(2)*^			
Number of hard stones^(3)^	6 (*n* = 30)	4 (*n* = 25)	*p* = 0.50
*Stone position*			
Number of lower pole stones	22 (39%)	22 (39%)	*p* = 1
Number of pyelum and PUJ stones	5 (9%)	8 (14%)	*p* = 0.37
Number of upper pole stones	18 (31%)	8 (14%)	*p* = 0.02
Number of midpolar stones	12 (21%)	19 (33%)	*p* = 0.14
*Stone density (HU)* ^*(4)*^			
Mean stone density (HU)	665 (*σ* = 160, *n* = 31)	740 (*σ* = 223, *n* = 29)	*p* = 0.10
Maximal stone density (HU)	965	1213	NA

Operator-related factors			
Experience with both techniques	2 investigators with equal experience		NA

Patient-related factors			
Mean BMI (kg/m^2^)	27 (*σ* = 3,8)	29 (*σ* = 3,3)	*p* = 0.001
Mean skin-to-stone distance (mm)^(5)^	90 (*σ* = 13, *n* =31)	104 (*σ* = 14, *n* = 29)	*p* < 0.001

Technique-related factors			
Shock frequency (bpm)	60		*p* = 1
Number of shocks	2500		*p* = 1
Ramping protocol	Identical in both groups		NA
Mean energy (Joule)	49 (*σ* = 11)	53 (*σ* = 11)	*p* = 0.002

*Study outcomes*			

Clinical outcome			
Number of stone-free patients	30	24	Noninferior^(6)^
Number of patients with asymptomatic residual fragments	15	16	
Number of patients with a positive outcome^(7)^	45	40	Noninferior^(6)^
Number of patients needing additional therapy	6	9	
Number of patients with no effect	6	8	
Number of patients with a negative outcome^(8)^	12	17	Noninferior^(6)^

Number of ESWL sessions			
Mean number of SWL sessions/patient	1.6 (*σ* = 0,7)	1.7 (*σ* = 0,7)	*p* = 0.431

Positioning			
Mean number of position changes/patient	8 (*σ* = 2)	6 (*σ* = 2)	*p* = 0.008
Mean time to position/patient	9 (*σ* = 2)	5 (*σ* = 2)	*p* < 0.001

Radiation			
Mean DAP (mGy/cm2)/SWL session	0	3005 (*σ* = 601)	*p* < 0.001

(1) Measured as longest stone distance on RX before the first SWL session. (2) Only 55/114 (48%) patients analysed their stone. (3) Calcium oxalate monohydrate (whewellite), cystine, or calcium phosphate (brushite) stones. (4) Hounsfield units as measured on CT abdomen. (5) Measured vertically in the US group and 45° in the FS group. (6) With a noninferiority margin of 10%. (7) Stone-free or asymptomatic residual fragments ≤ 4 mm. (8) Additional therapy or no effect.
